# Effects of Live Attenuated Vaccine and Wild Type Strains of *Edwardsiella ictaluri* on Phagocytosis, Bacterial Killing, and Survival of Catfish B Cells

**DOI:** 10.3389/fimmu.2019.02383

**Published:** 2019-10-09

**Authors:** Adef O. Kordon, Safak Kalindamar, Kara Majors, Hossam Abdelhamed, Wei Tan, Attila Karsi, Lesya M. Pinchuk

**Affiliations:** ^1^Department of Basic Sciences, College of Veterinary Medicine, Mississippi State University, Mississippi State, MS, United States; ^2^Department of Molecular Biology and Genetics, Faculty of Art and Sciences, Ordu University, Ordu, Turkey

**Keywords:** *Edwardsiella ictaluri*, live attenuated vaccines, catfish B cells, phagocytosis, bacterial killing, apoptosis

## Abstract

*Edwardsiella ictaluri*, a Gram-negative facultative intracellular pathogen, is the causative agent of enteric septicemia of catfish (ESC). The innate functions of B cells have been demonstrated in several teleost fish, including zebrafish, rainbow trout, and channel catfish. Recently, our group has developed several protective *E. ictaluri* live attenuated vaccines (LAVs). However, the innate role of catfish B cells to phagocytose and destroy *E. ictaluri* wild-type (WT) and live attenuated vaccine (LAV) strains has not been evaluated. In this study, we assessed the efficacy of *E. ictaluri* WT and two LAVs on phagocytosis, microbial killing, and survival of catfish anterior kidney (AK) B cells. Initially, we documented active uptake of *E. ictaluri* WT and two LAVs in B cells by flow cytometry and light microscopy. Then, we observed the *E. ictaluri* strains-induced phagosome and/or phagolysosome formation in the cytoplasm of catfish magnetically sorted IgM^+^ B cells. Furthermore, we demonstrated that AK B cells were able to destroy the internalized *E. ictaluri* WT and LAV strains efficiently. Finally, we documented early and late apoptotic/necrotic manifestations induced by *E. ictaluri* in catfish AK B cells. In conclusion, our results suggest that both LAVs and WT strain initiate similar innate immune responses such as active phagocytic uptake, induced bactericidal activity as well as promote early and late apoptotic changes in catfish B cells. Our data suggest that phagocytic and microbicidal B cells may serve as professional APCs in initiation of protective adaptive immune responses against ESC in channel catfish.

## Introduction

The primary function of B cells in the humoral branch of adaptive immunity is to secrete antibodies of increasing affinity and maintain an immunological memory (1). In recent years, it has been determined that B cells can be subdivided into different subsets with distinct morphology, phenotypes, and functional features and also contribute to innate immune responses. The recent discovery that live *Salmonella typhimurium* was engulfed by primary human B cells via B cell receptor (BCR)-dependent manner broke the long-held paradigm that B cells were unable to uptake large particulate antigens (2). Two subsets of mammalian B cells, marginal zone (MZ) and B-1 B cells, were classified as “innate B lymphocytes” based on their developmental, phenotypic, and functional characteristics contributing to innate immune responses, such as phagocytosis (3, 4). Importantly, phagocytic B cells from the peritoneal cavity were able to ingest bacteria, produce mature phagolysosomes, destroy the ingested bacteria and present the bacterial antigens to CD4^+^ T cells (5).

The first evidence on B cell phagocytosis in rainbow trout was reported by Li et al. (6). Like mammalian B-1 cells, B cells in teleost fish were able to engulf particles and kill the internalized pathogens (6, 7). However, teleost B cells were present in all systemic compartments including blood, spleen, and anterior kidney (AK) and representing 60% of all B cells. In contrast, phagocytic B cells in mammals were mainly found in the peritoneal cavity and represented a 30–40% of total B cell numbers (5, 6, 8–10). The ability of B cells to uptake soluble, particulate and bacterial antigens by phagocytosis has been documented in zebrafish and Atlantic salmon (11, 12). Furthermore, the phagocyting B cells that possessed phagolysosomes were described in rainbow trout suggesting their essential role in bacterial killing (6, 13). Additionally, B cells in Atlantic cod had higher phagocytic capacity to uptake fluorescent beads compared to neutrophils (12). Also, contrary to other teleost fish, the large amount of phagocytic B cells has also been found in catfish blood (6, 14).

*Edwardsiella ictaluri* is a Gram-negative facultative intracellular fish pathogen that causes enteric septicemia of catfish (ESC), which is one of the most devastating diseases in the US catfish industry (15–18). A live *E. ictaluri* vaccine (Aquavac-ESC) against ESC was developed by Klesius and Shoemaker (19), and this vaccine protected juvenile catfish (19). Then, immersion studies demonstrated that *E. ictaluri* LAVs stimulated protective immunity in catfish fry, fingerlings, and eyed catfish eggs (20–23). Recently, a live attenuated *E. ictaluri* isolate (S97-773) was developed by Wise, and oral vaccination with this isolate protected catfish fingerlings (24).

*Edwardsiella ictaluri* can survive and replicate in channel catfish macrophages, and *E. ictaluri* LAVs induced cell-mediated immunity to protect catfish against ESC (25–27). Also, catfish vaccinated with LAVs triggered humoral immune responses which augmented the bacterial killing activity of macrophages (25–27).

Recently, we demonstrated the phagocytic and killing properties of catfish peritoneal macrophages induced by two novel *E. ictaluri* LAV strains (*Ei*Δ*evpB* and ESC-NDKL1) developed in our laboratory which provided significant protection against ESC in both catfish fry and fingerlings (27–31). *Ei*Δ*evpB* was constructed by in-frame deletion of the *evpB* gene, one of the main components of type six secretion system (T6SS) (14). ESC-NDKL1 (Δ*gcvP*Δ*sdhC*Δ*frdA*) was constructed by in-frame deletion of three genes in the tricarboxylic acid cycle (*sdhC* and *frdA*) and one-carbon metabolism (*gcvP*) (30, 31). However, the roles of these LAVs on the phagocytosis and intracellular killing properties in catfish B cells were still unexplored. Therefore, the purpose of this study was to assess the ability of channel catfish AK B cells to phagocytose and kill LAV and WT strains of *E. ictaluri*. Increased phagocytic and killing ability of catfish B cells will delineate the role of B cells in innate immune responses in *E. ictaluri* infection.

## Materials and Methods

### Animals

Specific pathogen free (SPF) channel catfish were obtained from the fish hatchery at the College of Veterinary Medicine, Mississippi State University. All fish experiments were carried out based on a protocol approved by the Mississippi State University Institutional Animal Care and Use Committee (IACUC). Fish were maintained at 25–28°C throughout the experiments. To sedate and euthanize the catfish, tricaine methanesulfonate (MS-222, Western, Chemical, Inc.) was used. Samples were obtained as described below.

### Bacterial Strains and Opsonization

Bacterial strains for this study are listed in Table 1. *E. ictaluri* 93–146 wild-type (WT) and two LAVs strains were cultured in BHI agar or broth (Difco, Sparks, MD, United States), and incubated at 30°C for overnight. Two LAVs and WT strains were labeled with bioluminescence by transferring pAK*gfplux*1 from an *E. coli* donor strain (SM10λ*pir*) by conjugation as described previously (33). Ampicillin (Amp: 100 mg/ml), and colistin sulfate (Col: 12.5 mg/ml, Sigma–Aldrich, St. Louis, MN, United States) were added to media when they are required. *E. ictaluri* WT was incubated in the presence of 10% normal catfish serum for 30 min at room temperature.

**Table 1:** Bacterial strains and plasmids.

**Bacterial strain**	**Strains**	**References**
*Edwardsiella ictaluri* 93–146	Wild type; pEI1^+^; pEI2^+^; Col^r^	(32)
*Ei*Δ*evpB*	93–146 derivative;pEI1^+^; pEI2^+^; Col^r^;ΔevpB	(31)
ESC-NDKL1	93-146 derivative; pEI1^+^; pEI2^+^; Col^r^; ΔgcvPΔsdhCΔmdh	(30)

### Cell Preparation

Channel catfish (150–200 g) were used in this study. Anterior kidneys (AK) were dissected from 5 catfish and placed in a sterile culture dish that contained Phosphate-buffered saline (PBS). Tissues were pooled and crashed by using sterile forceps and passed through cells dissociation sieves (Sigma, St. Louis, MO) to obtain a single-cell suspension of AK. After that, cells were resuspended and washed in PBS. Cell suspensions were layered on Histopaque 1077 (Sigma) and centrifuged at 500 *g* for 30 min to obtain enriched white mononuclear cells (WMCs). Following centrifugation, WMCs were collected from the interface and washed three times in PBS at 500 *g* for 10 min. Cells were counted and assessed for viability by using a hemocytometer and trypan blue exclusion. Finally, cells were used for phagocytosis assessment.

### Phagocytosis and Flow Cytometry

Mononuclear white blood cells from AK were resuspended in L-15 medium (ThermoFisher Scientific) as described previously (11) and stained with primary monoclonal antibodies (mAbs, clone 9E1) to catfish IgM^+^ B cell-specific marker at 4°C (34, 35) followed by the addition of isotype-specific fluorochrome (R-PE) conjugate (Mouse F (ab) 2 IgG (H+L). (R-PE), R&D Systems, Inc.). After the staining procedure, cells were washed three times with PBS (all the steps were performed at 4°C in the dark).

Following the staining, AK WMCs were resuspended in L-15 medium and 2 × 10^6^ cells per well were transferred into 6-well plates (FisherScientific, Pittsburgh, PA, United States). Green Fluorescence Protein (GFP) transformed bacterial strains were added in 1:50 ratio to each well and incubated at 30°C in the dark for 30 min to determine the phagocytic ability of catfish B cells.

**Figure 1:**
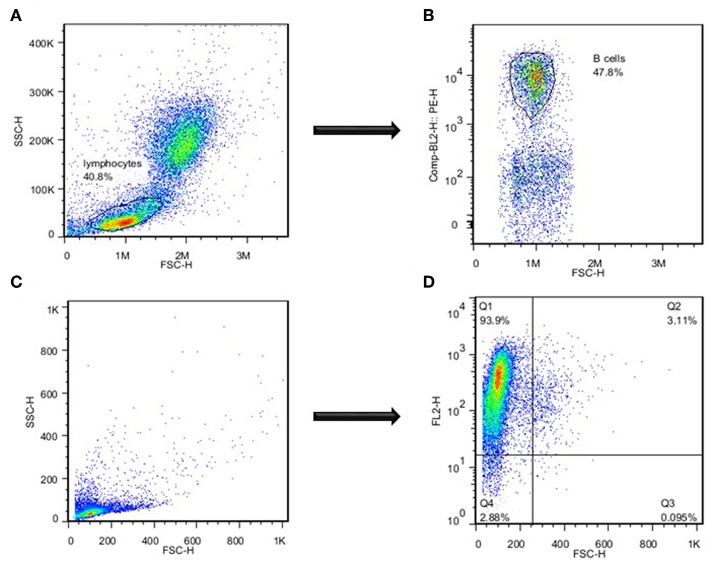
Separation of catfish B cells by flow cytometry. **(A)** Assessment of lymphocytes based on their size and granularity. **(B)** Identification of B cells based on the intensity of B cell-specific staining with mAbs specific to channel catfish IgM^+^ B cells. **(C)** Assessment of highly enriched magnetically sorted IgM^+^ cell population based on their size and granularity. **(D)** Identification of B cells based on the intensity of catfish B cell-specific staining. One of three representative experiments.

**Figure 2:**
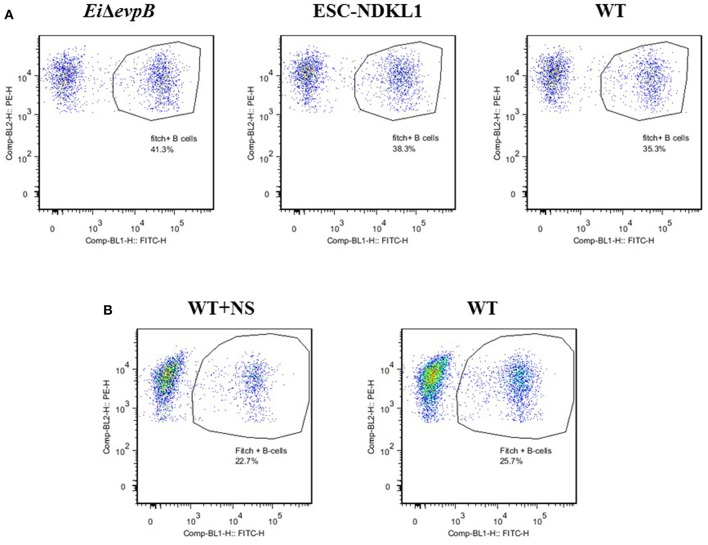
Active uptake of *E. ictaluri Ei*Δ*evpB*, ESC-NDKL1, WT **(A)** and WT opsonized with normal serum **(B)** strains in catfish B cells. B cells were shown by flow cytometry-two color with Dot Plot statistics. Dots in the circles indicate phagocytic B cells for each group. Data represent one of three biological replicas from the AK –derived mononuclear cells combined from five fish.

Catfish WMCs and lymphocytes were gated based on their relative size and granularity by using forward and side scatters, FSC and SSC, respectively (Figure 1A). After setting a gate on IgM^+^ cells (Figure 1B), phagocytic B cells were determined based on the intensity of GFP fluorescence (Figure 2). The percentage of phagocytic B cells was determined by NovoCyte Flow Cytometry (ACEA Biosciences, Inc.) using two-color analysis with Dot Plot Quadrant statistics. Samples were analyzed using FlowJo 7.6.4 Software (Tree Star Inc.).

### Cell Sorting

IgM^+^ B cells were positively selected by magnetic sorting from AK WMC populations as described previously with minor modifications (11). Briefly, WMCs were obtained from AK by using Histopaque 1077 separation, resuspended in L-15 medium and passed through the pre-separation filters (Miltenyi Biotec) to remove cell clumps. Then, mAbs specific to channel catfish IgM^+^ B cells were used to positively identify catfish B cells (34, 35). Followed by incubation on ice for 30 min, cells were washed and resuspended in MACS buffer (Miltenyi Biotec), and anti-mouse IgG (H+L)-magnetic microbeads (Miltenyi Biotec) were added to cell suspensions, incubated at 4°C for 15 min in the dark, washed and transferred onto a LS separation column (Miltenyi Biotec), according to manufacturer instructions. The purity of the resulting low size/low granularity IgM^+^ populations was 85–94% by flow cytometry (Figures 1C,D). After magnetic separation, positively selected B cells were cultured in the L-15 medium at 28°C overnight to detach the magnetic microbeads. Followed by Histopaque 1077 separation and assessment of viability of the resulting B cell populations by trypan blue exclusion (1–3%), the purity of the sorted IgM^+^ B cells was determined by a FACSCalibur Flow Cytometer (Becton Dickinson), and highly enriched by sorting IgM^+^ B cells were used to assess the bacterial killing ability, morphology and apoptotic changes.

### Cytospin and Light Microscopy

Highly enriched IgM^+^ and IgM^−^ cell populations were incubated in the presence of *E. ictaluri* strains to characterize their morphology and phagocytic capacity. Following the incubation, cells were harvested and washed in PBS. Then, the cytospins were prepared at 500 rpm for 1 min by using a Cyto-Tek centrifuge machine. All samples were fixed on the slides, and Giemsa staining procedure (May-Grunwald Procedure) was applied to observe the morphology of IgM^+^ cell population. The Wright's stain (Hemacolor, Merck) was applied to observe the IgM^−^ cell population morphology as described previously (36). Samples were analyzed with an Olympus BX60 microscope (OlympusU-TV1 X) and photographed by using Infinity software (Lumenera Corporation).

### Bacterial Killing Assay

The bacterial killing assay was performed as described previously with some minor modifications (26, 37). Briefly, sorted B cells were resuspended with L-15 medium supplemented with 10% FBS, 1% L-glutamine, and 1.5% HEPES buffer and 0.5 × 10^6^ cells per well were transferred to 96-well plates (Evergreen Scientific). Then, WT and two LAVs strains were added to cell suspension in 1:20 ratio that did not affect the viability of B cell populations by trypan blue exclusion (1–5%) for 48 h in culture compared to uninfected controls. Plates were centrifuged at 1,500 rpm for 5 min at room temperature to compact cells and bacteria and incubated at 30°C for 30 min. After that, plates were centrifuged at 2,000 rpm for 7–10 min to remove the supernatant. Next, the pellet was resuspended with L-15 medium supplemented with 10% FBS, 1% L-glutamine, 1.5% HEPES buffer, and 100 μg/ml gentamicin (Gibco, Life Technologies, Grand Island, NY, United States) to kill extracellular bacteria, and incubated at 30°C for 1 h. Following the killing of extracellular bacteria, plates were washed in PBS and resuspended in L-15 medium containing 10 μg/ml gentamicin (time 0) for 48 h incubation with 5% CO_2_ at 30°C in the black 96-well plates (Fisher Scientific) to determine the number of the live intracellular *E. ictaluri* in catfish B cells. Statistical analysis was acquired with the results obtained from Cytation 5 Cell Imaging Multi-Mode Reader (BioTek).

### Apoptosis Assay

The apoptosis assay was performed as described previously with minor modifications (38). Positively selected B cells were resuspended in L-15 medium and 2 × 10^6^ cells per well were transferred to 24-well plates (Tissue Culture Plate, CELLTREAT). Then, WT and LAVs *E. ictaluri* strains were added to the plates in 1:50 ratio and incubated at 30°C in the dark for 30 min and 3 h to detect early and late apoptosis in catfish B cells. After incubation, cells were collected and washed with cold PBS by centrifugation at 4°C. Apoptosis in catfish B cells was assessed by using Annexin V-FITC Apoptosis Kit according to manufacturer's instructions (BioVision, Inc., Mountain View, CA). Briefly, cells were resuspended in 1x Binding buffer and incubated with Annexin-V-FITC and propidium iodide (PI) for 5 min at room temperatures in the dark. Samples were analyzed by NovoCyte Flow Cytometry using two-color analyses with Dot Plot Quadrant Statistics. Also, staurosporine (10 μM, Sigma)-treated catfish B cells were used as positive control for apoptosis.

### Statistical Analysis

One-way ANOVA with PROC GLM procedure in SAS (v 9.4, SAS Institute, Inc., Cary, NC) was used for the bioluminescence intensity of GFP-labeled *E. ictaluri* strains of antigen uptake and bacterial killing assay. Separate models were fit for each time point. The fixed effect for the bioluminescence intensity of GFP-labeled *E. ictaluri* strains was treatment. LSMEANS statements with TUKEY adjustment was used to evaluate significant differences among treatments for each model. An alpha level of 0.05 was used to determine statistical significance. The distribution of the residuals was evaluated for each model to make sure the assumptions of normality and homoscedasticity for the statistical method had been met.

Linear models with PROC MIXED in SAS for Windows 9.4 were applied for the percentage of live cells, early apoptotic cells, late apoptotic cells, and necrotic cells. Fixed effects for each outcome (live cells, early apoptotic cells, late apoptotic cells, and necrotic cells) of apoptosis assay were treatment, hour and their interaction. In the case of a significant interaction term, differences in least squares means between 0.5 and 3 h for each of the treatments and also between treatments for each hour were calculated by using an LSMESTIMATE statement. Also, the simulate adjustment for multiple comparisons was used in the case of the significant terms.

The level of significance for all tests was set at *P* < 0.05. The distribution of the residuals was evaluated for each model to make sure the assumptions of normality and homoscedasticity for the statistical method had been met.

## Results

### Active Phagocytic Uptake of *E. ictaluri* WT and LAVs Strains in AK B Cells

In this study, we determined the active uptake of *E. ictaluri* WT and two LAVs strains in catfish B cells (Figure 2A). Separated from PBMC by two-color flow cytometry IgM^+^ B cells actively endocytosed *E. ictaluri* LAV and WT strains (Figure 2A), and the phagocytic intensity levels of the LAV and WT strains of *E. ictaluri* in catfish AK B cells of three biological replicas did not differ significantly (*P* > 0.05, data not shown). Furthermore, we used an additional protocol for active bacterial uptake assessment in the AK B cells exposed to the WT *E. ictaluri* strain opsonized with normal catfish serum (Figure 2B). Separated by flow cytometry B cells actively endocytosed opsonized bacterial strain (Figure 2B). The data represent one of three biological replicas from the AK–derived mononuclear cells combined from five fish.

**Figure 3:**
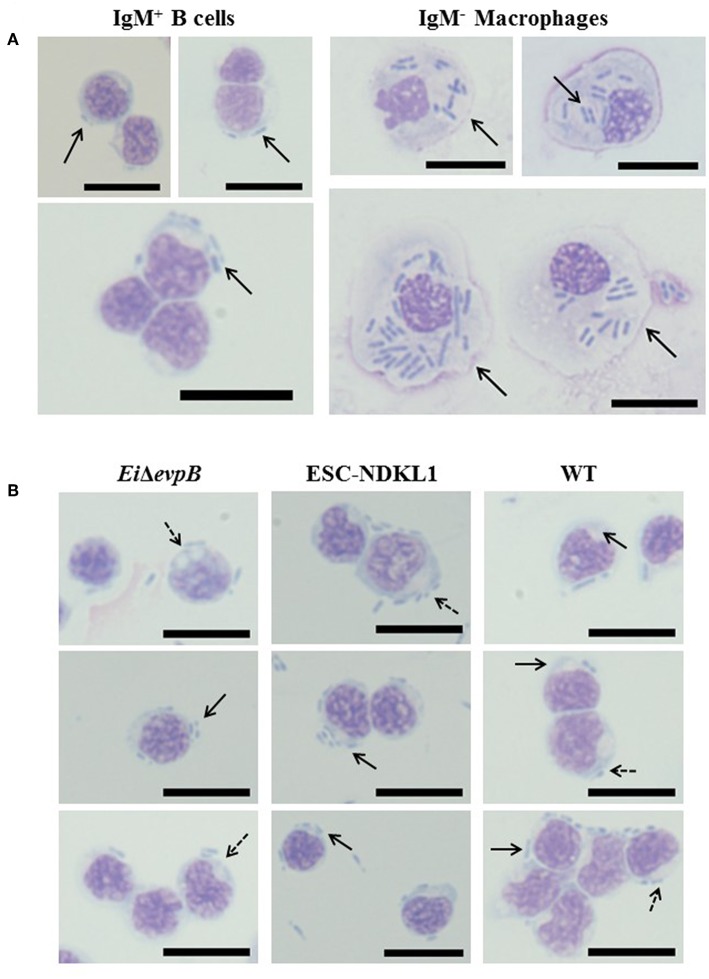
**(A)** Uptake of *E. ictaluri* WT strain in AK IgM^+^ B cells and AK macrophages (IgM- adherent cells) by light microscopy. Phagocytosis of WT strain in IgM^+^ B cells (93.9% purity, left column). Phagocytosis of *E. ictaluri* in IgM^−^ macrophages (right column). **(B)** Active uptake of *E. ictaluri* LAV and WT strains in highly purified catfish B cells (93.9% IgM^+^ cells) by light microscopy. Solid arrows indicate the engulfed bacterial strains by catfish AK B cells (*Ei*Δ*evpB*, ESC-NDKL1, and WT shown in left, middle and right columns, respectively). Arrows with dots indicate phagosomes and/or phagolysosomes in the cytoplasm of catfish B cells. 100 × magnification, scale bar 20 micrometers.

To confirm active phagocytic uptake in catfish B cells by flow cytometric approach, we assessed *E. ictaluri* WT and two LAVs strains uptake in highly purified magnetically sorted IgM^+^ B cells by light microscopy (Figure 3). In addition to the apparent intracellular bacterial uptake of LAVs and WT *E. ictaluri*, the phagosome and/or phagolysosome formation was evident in the cytoplasm of catfish B cells (Figure 3). To characterize the suggested contrasting morphology and phagocytic capacity of the unlabeled (IgM^−^) cell populations, we assessed their incorporation of *E. ictaluri* WT strain by light microscopy (Figure 3). The IgM^−^ cell populations showed typical macrophage morphology such as larger size and cytoplasm presence with dramatically increased numbers of the engulfed bacteria compared to the sorted IgM^+^ B cells (Figure 3).

### Killing of *E. ictaluri* and LAVs Strains by Catfish B Cells

**Figure 4:**
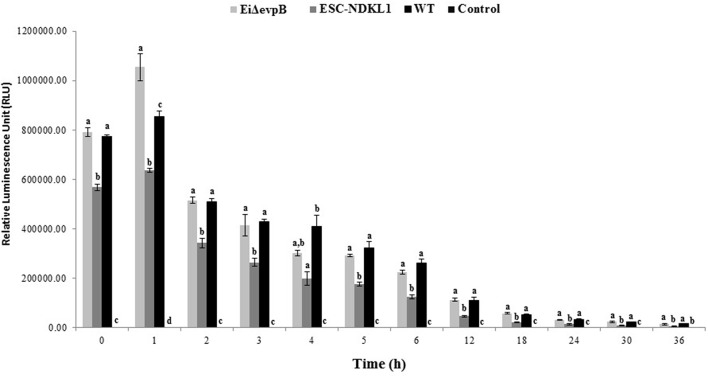
Bacterial killing of *E. ictaluri* WT and LAVs strains by catfish B cells. Letters show significant differences between the treatments (at any given time point, *P* < 0.05). One way ANOVA with PROC GLM procedure in SAS for 9.4 was used for bacterial bioluminescence intensity. The data represent the mean of four biological replicas of the AK– derived B cells combined from five fish ±SD in each experimental group.

**Figure 5:**
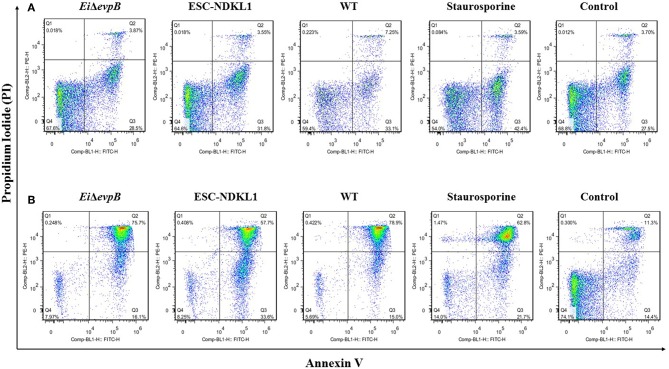
The effects of *E. ictaluri* WT and LAVs strains on catfish AK B cell apoptosis. Early and late apoptotic changes in catfish magnetically sorted B cells exposed to *Ei*Δ*evpB*, ESC-NDKL1, and WT *E. ictaluri* strains at 30 min **(A)** and 3 h **(B)**. Necrotic cells (Q1, PI+/Annexin V-); late apoptotic cells (Q2, PI+/Annexin V+); early apoptotic cells (Q3, Annexin V+/PI-); live cells (Q4, PI-/Annexin V-). The data represent one of three biological replicas of AK-derived B cells combined from five fish in each experimental group.

After determining the levels of phagocytosis of *E. ictaluri* and LAVs strains in catfish B cells, we examined how effective catfish B cells were at destroying the ingested bacteria at 30°C by applying more sensitive Cell Imaging technology (Figure 4). IgM^+^ B cells were positively selected by magnetic sorting from AK WMC populations (Figures 1A–D). WMCs and lymphocytes were gated based on their relative size and granularity by using forward and side scatters, FSC and SSC, respectively (Figure 1A). After setting a gate on IgM^+^ cells (Figure 1B), mAbs specific to channel catfish IgM^+^ B cells were used to positively identify catfish B cells (Figure 1C), The purity of the resulting IgM^+^ populations was 85–94% by flow cytometry (Figure 1D). The initial intensity of the intracellular bacterial luminescence (time 0) did not show significant differences in B cells challenged with *Ei*Δ*evpB* and WT strains. However, the WT strain intensity was significantly higher compared to the luminescence intensity of ESC-NDKL1 in B cells (Figure 4). Negative control B cells not exposed to bacteria showed the background low levels of luminescence intensity (Figure 4). Significant differences in the intensity of luminescence between all challenges were documented at 1 h post-incubation (Figure 4). For example, the intensity of *Ei*Δ*evpB* luminescence in B cells at this time was significantly higher than the luminescence of WT and ESC-NDKL1 strains (Figure 5). Moreover, the luminescence of WT strain in B cells was significantly higher than ESC-NDKL1 luminescence (Figure 4). Interestingly, the luminescence of both LAVs and WT strains in catfish B cells significantly decreased at 2 h; however, there were significant differences between the challenges and negative control B cells not exposed to bacteria (Figure 4). The luminescence intensity of all *E. ictaluri* strains showed time-dependent gradual decreases, and there were no significant differences in the luminescence of ESC-NDKL1 and negative control group at 36 h in catfish B cells (Figure 4). However, the intensity of WT and *Ei*Δ*evpB* luminescence at 36 h was still significantly higher compared to the group challenged with ESC-NDKL1 and negative control group (Figure 4). Finally, there were significant decreases in the luminescence of *Ei*Δ*evpB* after 2 and 3 h of incubation compared to non-significant changes in the luminescence of ESC-NDKL1 and WT in catfish B cells at these time points (*P* < 0.001). Time-dependent significant decreases in bacterial luminescence were documented for both LAVs and WT strains, however more than 50% of the initial *Ei*Δ*evpB* uptake was eliminated at 4 h, ESC-NDKL1 uptake at 3 h and the WT uptake at 5 h post exposure (Supplemental Figure 1). Our data showed that catfish B cells were capable of killing *E. ictaluri* WT and both LAVs strains; however, they were more efficient at killing of ESC-NDKL1 than destroying of WT and *Ei*Δ*evpB* strains.

### Early and Late Apoptotic Changes in Catfish B Cells Exposed to WT *E. ictaluri* and LAVs

**Figure 6:**
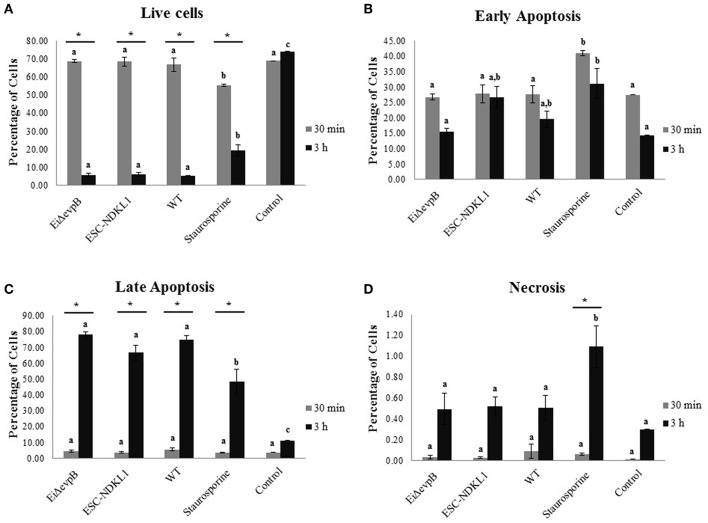
Statistical interpretation of early and late apoptotic changes in catfish AK B cells. Live B cells **(A)**, early apoptotic cells **(B)**, late apoptotic cells **(C)**, and necrotic cells **(D)**. Letters shows significant differences between treatments at a given time point. *Symbol indicates significant difference between 30 min and 3 h for each treatment (*P* < 0.05). Linear models with PROC MIXED in SAS for 9.4 were applied for all outcomes: the percentage of live cells, early apoptotic cells, late apoptotic cells, and necrotic cells. The data represent the mean of three biological replicas of the AK-derived B cells combined from five fish ±SD in each experimental group.

Early and late apoptotic changes (one of three biological replicas of AK-derived B cells combined from five fish in each experimental group) in B cells exposed to LAV and WT *E. ictaluri* strains have been assessed at 30 min (Figure 5A) and 3 h post-exposure (Figure 5B). Statistical analysis of early and late apoptotic, and necrotic changes in catfish AK B cells based on the three biological replicas is shown in Figures 6A–D. Interestingly, there were no significant differences in the percentages of live cells between B cells exposed to WT and LAV strains at both 30 min and 3 h incubation times, however as expected, the percentages of live B cells treated with staurosporine (positive control) were significantly decreased compared to other groups (Figure 6A). Also, there were significant differences in the percentages of live B cells between 30 min and 3 h incubation times with decreased percentages of live cells at 3 h post-treatment (Figure 6A). Furthermore, there were no significant differences in the percentages of early apoptotic cells between treatments exposed to LAVs and WT strains at 30 min; however, staurosporine induced significantly higher levels of early apoptosis at 30 min compared to other groups (Figure 6B). In addition, there were no significant differences in the percentages of early apoptotic cells between the groups exposed to staurosporine, WT and ESC-NDKL1 strains at 3 h post-treatment (Figure 6B). In contrast, *Ei*Δ*evpB* caused significantly less early apoptotic changes than staurosporine at 3 h at this time (Figure 6B). Moreover, there were no significant differences in the percentages of early apoptotic cells between 30 min and 3 h incubation times (Figure 6B). The percentages of late apoptotic cells at 3 h incubation time significantly increased in all treatments except negative control (Figure 6C). There was no significant difference in the percentages of late apoptotic cells between treatments at 30 min. However, the percentages of late apoptotic cells in the groups exposed to WT and LAV strains were significantly higher than in the group treated with staurosporine at 3 h (Figures 6C,D). There were no significant differences in the percentages of necrotic cells between treatments at 30 min; however, the percentages of necrotic cells at 3 h significantly increased in the group exposed to staurosporine only (Figure 6D).

## Discussion

B cells possess the ability to capture antigens, process into peptides, and load the peptides onto MHC class II molecules for their presentation to CD4^+^ T cells (39). Several studies reported that B cells in teleost fish served as professional antigen presenting cells (APCs). For instance, zebrafish B cells were able to present both soluble and particulate antigens to prime naïve CD4^+^ T cells. Also, this study showed that the expression of MHC class II molecules and co-stimulatory molecules (CD86 and CD83) was upregulated in B cells during the presentation of antigens. Several studies have demonstrated that B cells in teleost fish were important APCs that activated T cells and initiated adaptive immunity *in vivo* and *in vitro* (11, 40, 41). Phagocytosis and intracellular killing activities are crucial properties of all professional APCs. Therefore, the current research aimed to determine the phagocytic and bactericidal activity of catfish AK B cells in the uptake of *E. ictaluri* WT and two LAV strains developed in our laboratory. In teleost fish, AK has a vital function as a hemopoietic organ, which produces all blood elements (42–45). Several studies reported that AK is one of the target organs in early of *E. ictaluri* infection. For instance, leukocytes including *E. ictaluri* were observed in the AK of channel catfish at 48 h post-infection (46). Also, bioluminescent *E. ictaluri* dispersion was found in the catfish AK at 15 min after intraperitoneal injection (47). Recently, the *E. ictaluri*-induced necrosis was demonstrated in the hemopoietic tissue part of catfish AK (48).

We documented phagocytic capability mediated through non-opsonic and opsonic receptors and bacterial killing activity of B cells in channel catfish against intracellular pathogens *in vitro* confirming the data obtained in the previous studies. First, we demonstrated that catfish B cells were able to engulf *E. ictaluri* WT and two LAV strains. Also, we documented phagocytic uptake of the WT strain opsonized with normal catfish serum. Notably, numerical but not significant increases in the intensity of phagocytic uptake have been documented by flow cytometry in the AK B cells exposed to *Ei*Δ*evpB* and WT strains compared to their counterparts challenged with ESC-NDKL1 LAV. Similarly, our previous data revealed that the intensity of *Ei*Δ*evpB* LAV phagocytic uptake was significantly higher compared to the ESC-NDKL1 strain in catfish peritoneal macrophages (27). However, significant differences in bacterial uptake between two LAVs and WT strains were evident in the bacterial killing assay after removal of all the attached bacterial cells. In addition, bacterial killing of the *Ei*Δ*evpB* LAV was significantly increased compared to bacterial killing of ESC-NDKL1 and WT strains in catfish B cells after 2 and 3 h of incubation. Phagocytic ability of B cells has been shown in several teleost fish, such as zebrafish, rainbow trout, Atlantic salmon and Atlantic cod (6, 11, 12). In addition, B-1 and MZ subsets of B cells in mammals were capable of uptake of pathogens (3). Moreover, a recent study in rainbow trout demonstrated that fish IgM^+^ B cells share common phenotypic and functional characteristics of mammalian B1 cells (49).

Professional phagocytic cells, such as macrophages, recognized and engulfed pathogens into vesicles known as phagosomes that fuse with lysosomes to form phagolysosomes (50, 51). Ingested pathogens were destroyed and killed in phagolysosomes by enzymes and antimicrobial substances, such as NO (52). Like professional phagocytes, murine B-1 cells have been shown to mature their phagosomes into phagolysosomes (5). In this study, we observed phagosome and/or phagolysosome formation in the cytoplasm of catfish B cells by light microscopy. However, the size of phagosome and/or phagolysosome in AK macrophages was larger than those found in B cells. Moreover, ingested bacteria numbers in the phagosomes of AK macrophages were virtually higher than the numbers of bacteria in the B cell phagosomes confirming our recent report on WT and LAV *E. ictaluri* strains detected in the phagosomes of peritoneal macrophages in catfish (27). Furthermore, our data showed that catfish B cells were capable of destroying WT and LAV *E. ictaluri* strains. Kinetics of bacterial killing in catfish B cells were distinct than the kinetics described in peritoneal macrophages. The numbers of ingested bacteria decreased significantly in peritoneal macrophages at 10 h incubation period (27). However, the luminescence of internalized *E. ictaluri* significantly declined in catfish B cells after 2 h of incubation that correlated with increased numbers of early and late apoptotic and necrotic AK B cells between 30 min and 3 h of incubation. Although the microbicidal capacity of B cells was limited compared to peritoneal macrophages in catfish, the rapid destruction of bacterial antigens in B cells could provide early activation cues to specific T cells against ESC. Similar to mammals, internalization of particles by phagocytic B cells in teleost fish induced the formation of phagolysosomes with the fusion of lysosomes to phagosomes and exhibited the capability to kill ingested particles (6, 13). Another study in rainbow trout showed that sorted IgM^+^ and IgT^+^ B cells were able to kill internalized bacteria, *Escherichia coli* (9). Also, phagolysosome formation in mammalian B cells supported the killing activity of phagocytic B cells. B cells from the peritoneal cavity of mice engulfed *Staphylococcus aureus* (*S. aureus*), and uptake of bacteria led to the formation of phagolysosomes followed by activation the degradation pathways to kill the ingested bacteria (53).

Apoptosis, the process of programmed cell death, is identified by distinct morphological changes and biochemical modifications, such as protein cleavage and DNA fragmentation (54–56). Apoptosis is a crucial component of numerous processes including development, normal cell turnover, and the immune system, and this process occurs during normal development and aging (54). Also, apoptosis occurs in response to diverse physiological and pathophysiological stimuli and diseases (57, 58). In this study, we applied an apoptosis assay to detect early and late apoptotic changes in catfish B cells exposed to *E. ictaluri* WT and two LAV strains. Our data showed that *E. ictaluri* strains caused early and late apoptosis in catfish B cells. It was demonstrated previously that a few numbers of mouse B cells from peritoneal cavity incubated with *S. aureus* underwent apoptosis (53). Moreover, *Trypanosoma brucei* induced the loss of IgM^+^ B cell population in mice by causing apoptosis (59). In addition, *Mycoplasma bovis* induced apoptosis of lymphocytes in the bovine model (60).

In conclusion, our study demonstrated that efficacious *E. ictaluri* LAVs facilitate the phagocytic activity and effective killing of internalized bacteria in channel catfish B cells. We also documented enhanced phagocytic and microbicidal ability in catfish B cells exposed to the *Ei*Δ*evpB* LAV compared to the ESC-NDKL1 counterpart. For the first time, we documented the presence of phagosome and/or phagolysosome formations and the engulfed bacteria in AK B cells of catfish. These results suggest that both LAVs exploit similar to WT *E. ictaluri* innate immunological mechanisms such as active phagocytic uptake, bactericidal activity and promote early and late apoptotic changes in catfish B cells. However, further research is needed to assess the role of B cells in professional antigen presentation of bacterial-derived peptides to specific T cells and activation of protective adaptive immune responses against ESC in channel catfish. Although identification of non-opsonic receptors recognizing and binding to chemical structures on the surface of bacteria involved in the phagocytic uptake was beyond of the scope of this study, further research should address the molecular mechanism of receptor-mediated endocytosis of *E. ictaluri* as well as other intracellular pathogens in catfish B cells.

## Data Availability Statement

All datasets generated for this study are included in the manuscript/Supplementary Files.

## Ethics Statement

All fish experiments were carried out based on a protocol approved by the Mississippi State University Institutional Animal Care and Use Committee.

## Author Contributions

LP and AK conceived and designed the experiments and provided the original idea of the study, and also contributed reagents, materials, and tools. AOK, SK, KM, HA, and WT performed the experiments. AOK wrote the first draft of the manuscript and was involved in all aspects of the study. All authors were involved in critical interpretation of the data, manuscript revision, and final version approval.

### Conflict of Interest

The authors declare that the research was conducted in the absence of any commercial or financial relationships that could be construed as a potential conflict of interest.

## Supplementary Material

Supplemental Figure 1 Kinetics of the engulfed *E. ictaluri* LAV and WT strains killing in catfish B cells. Letters show significant differences between the time points (*P* < 0.0001). One way ANOVA with PROC GLM procedure in SAS for 9.4 was used for bacterial bioluminescence intensity. Initial uptake of bacteria at time 0 was considered 100%. The data represent the mean of four biological replicas of the AK– derived B cells combined from five fish ±SD in each experimental group.

## Abbreviations

*E. ictaluri*: *Edwardsiella ictaluri*
ESC: Enteric septicemia of catfish
LAV: Live attenuated vaccine
WT: Wild-type
AK: Anterior kidney
SPF: Specific pathogen free
BHI: Brain heart infusion
APCs: Antigen-presenting cells.
