# Supplementary figures and images for: Effects of Live Attenuated Vaccine and Wild Type Strains of Edwardsiella ictaluri on Phagocytosis, Bacterial Killing, and Survival of Catfish B Cells
Source: Front Immunol. 2019 Oct 9;10:2383. doi: 10.3389/fimmu.2019.02383 (PMC6794446; doi:10.3389/fimmu.2019.02383)

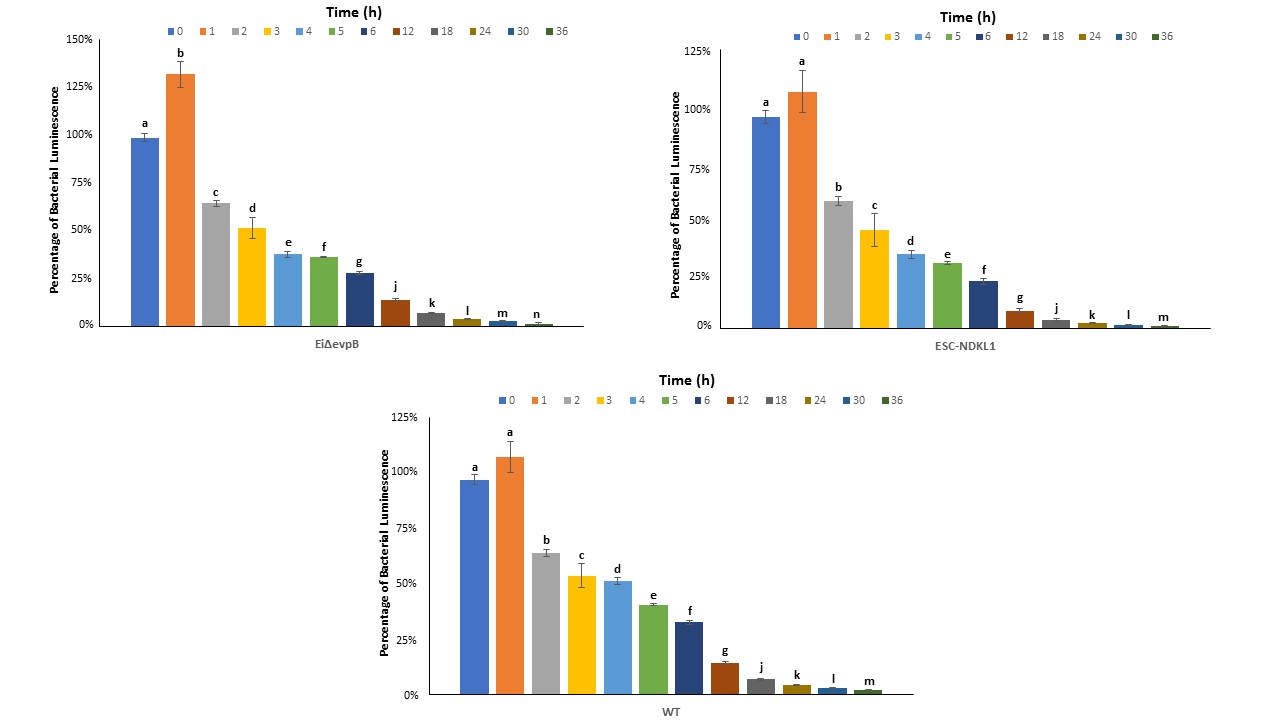

Supplement: Supplemental Figure 1 — Kinetics of the engulfed E. ictaluri LAV and WT strains killing in catfish B cells. Letters show significant differences between the time points (P < 0.0001). One way ANOVA with PROC GLM procedure in SAS for 9.4 was used for bacterial bioluminescence intensity. Initial uptake of bacteria at time 0 was considered 100%. The data represent the mean of four biological replicas of the AK– derived B cells combined from five fish ±SD in each experimental group. [file Image_1.JPEG]
